# Drinking behaviours and blood alcohol concentration in four European drinking environments: a cross-sectional study

**DOI:** 10.1186/1471-2458-11-918

**Published:** 2011-12-12

**Authors:** Karen Hughes, Zara Quigg, Mark A Bellis, Ninette van Hasselt, Amador Calafat, Matej Kosir, Montse Juan, Mariangels Duch, Lotte Voorham

**Affiliations:** 1 Centre for Public Health, Liverpool John Moores University, 15-21 Webster Street, Liverpool L3 2ET, UK; 2 Trimbos-instituut, Da Costakade 45, 3521 VS Utrecht, Netherlands; 3 European Institute of Studies on Prevention (IREFREA), Rambla 15, 07003 Palma de Mallorca, Spain; 4 Institute for Research and Development 'Utrip', Trubarjeva cesta 13, SI-1290, Grosuplje Ljubljana, Slovenia

## Abstract

**Background:**

Reducing harm in drinking environments is a growing priority for European alcohol policy yet few studies have explored nightlife drinking behaviours. This study examines alcohol consumption and blood alcohol concentration (BAC) in drinking environments in four European cities.

**Methods:**

A short questionnaire was implemented among 838 drinkers aged 16-35 in drinking environments in four European cities, in the Netherlands, Slovenia, Spain and the UK. Questions included self-reported alcohol use before interview and expected consumption over the remainder of the night. Breathalyser tests were used to measured breath alcohol concentration (converted to BAC) at interview.

**Results:**

Most participants in the Dutch (56.2%), Spanish (59.6%) and British (61.4%) samples had preloaded (cf Slovenia 34.8%). In those drinking < 3 h at interview, there were no differences in BAC by gender or nationality. In UK participants, BAC increased significantly in those who had been drinking longer, reaching 0.13% (median) in females and 0.17% in males drinking > 5 h. In other nationalities, BAC increases were less pronounced or absent. High BAC (> 0.08%) was associated with being male, aged > 19, British and having consumed spirits. In all cities most participants intended to drink enough alcohol to constitute binge drinking.

**Conclusions:**

Different models of drinking behaviour are seen in different nightlife settings. Here, the UK sample was typified by continued increases in inebriation compared with steady, more moderate intoxication elsewhere. With the former being associated with higher health risks, European alcohol policy must work to deter this form of nightlife.

## Background

Reducing the negative consequences of drinking and alcohol intoxication is a key global health priority [1]. The European Region has the highest levels of alcohol consumption in the world and the greatest proportion of ill health and premature death attributable to alcohol [2]. Although most alcohol-related deaths occur in older age groups, the burden of alcohol on mortality and morbidity falls disproportionately on young people, largely through acute alcohol-related injuries [3-5]. The relationship between alcohol and injury is dose-responsive, with injury risks increasing with blood alcohol concentration (BAC) [6] and being particularly acute for heavy episodic drinkers [7]. Studies show that heavy episodic drinking peaks in the late teenage years and early adulthood [8], with much alcohol use and harm in this age group taking place in public drinking environments [9]. Thus, policy recommendations to reduce harm from alcohol both internationally and in Europe are focusing on managing drinking environments, including through regulation, enforcement, management policies for bars and nightclubs, bar staff training and care for intoxicated individuals [1]. However, there are currently little empirical data available on drinking behaviours in European drinking environments to inform such measures.

There are wide variations in drinking cultures across Europe. Traditionally, a north-south gradient has been characterised by daily, moderate consumption of wine with food in Southern countries and infrequent heavy consumption of beer or spirits as an intoxicant in Northern countries [10]. Whilst cultural differences are still apparent [10-12], drinking patterns are thought to be converging across Europe. In particular, recent years have seen increases in heavy episodic drinking among young people in many European countries [13], with concerns that Northern cultures of heavy drinking and intoxication are spreading [4,14]. However, cross-national comparisons of alcohol use in young Europeans typically rely on school surveys of adolescents [13,15]. Data on drinking behaviours in young adults are drawn largely from general population surveys, that are known to underestimate consumption [16,17] and provide little context on nightlife drinking behaviours. A small number of cross-national studies have examined nightlife drinking behaviours and associated harms in young Europeans [11,12] with, for example, higher levels of drunkenness having been identified in those from Northern European countries [11]. However, few European studies have attempted to measure drinking behaviours actually in nightlife environments, and none have done so on a cross-national basis.

Recent single-country studies have used breathalyser tests alongside surveys to measure alcohol consumption and intoxication in nightlife settings [18-21]. In the UK, strong correlations have been found between reported alcohol use, BAC and intoxication in nightlife, with associations found between certain nightlife behaviours (e.g. preloading [consuming off-licensed alcohol before entering the nightlife environment]; staying out later) and higher levels of alcohol consumption and intoxication [18]. Here, we present findings from a study that used a similar methodology in nightlife environments in four European cities: Liverpool (UK); Ljubljana (Slovenia); Palma de Mallorca (Spain); and Utrecht (the Netherlands). While each city cannot be considered nationally representative, all are popular nightlife locations selected to be indicative of a range of nightlife cultures. Thus, UK nightlife is typically characterised by high levels of alcohol use, drunkenness and related harms including violence [22-24], while reports suggest that alcohol use and associated violence have also been increasing in Dutch nightlife settings [25]. The Balearic Islands in Spain have a long history of nightlife linked to international tourism; while heavy nightlife drinking has traditionally been limited to tourists, recent years have seen increasing alcohol use in local youth, linked to the practice of botellón--the gathering of young people in public places to consume off licensed alcohol (often before visiting bars and nightclubs) [26]. Research on nightlife in Slovenia is scarce; while levels of adolescent alcohol use are relatively high [27] little is known about nightlife drinking behaviours, although associated problems such as violence are considered rare [28].

The objectives of this study were to examine the amount of alcohol young adults reported drinking across the course of a night out in the four cities, and to measure BAC among drinkers during their night out. The study aimed to establish and test a cross-national methodology to measure drinking behaviours in nightlife environments, and to provide an initial assessment of variation in drinking patterns and intoxication across different European nightlife settings. Developing this knowledge is important in understanding differences in nightlife alcohol consumption across cultures and consequently in informing the development of appropriate and culturally relevant measures to reduce harm in drinking environments. Thus, analyses explore differences between city samples in reported drinking behaviours and BAC levels over a night out.

## Methods

The study took place in four cities: Utrecht (the Netherlands); Ljubljana (Slovenia); Palma de Mallorca (Spain); and Liverpool (UK). All sites were part of a broader investigation into alcohol-related harm in drinking environments [22]. At the time of this study^1^, Utrecht (population ~300,000) city centre had around 160 nightlife venues (pubs, bars and nightclubs), with most closing between 2 am and 5 am. Liverpool (population ~435,000) city centre had 304 nightlife venues, over half of which were licensed to stay open later than 2 am. Despite being the only capital city studied, Ljubljana (population ~277,000) had the smallest number of city centre nightlife venues (n = 41) that closed between midnight and 5 am. Palma (population ~400,000) reported 500 nightlife venues within its broader municipality area, including those in tourist resorts surrounding the city; however our study focused on city centre drinking environments popular with locals, where closing times were largely between 4 am and 6 am. In Liverpool, Ljubljana and Palma, the legal age for alcohol sales was 18. In Utrecht, beer and wine sales were permitted at age 16, with stronger alcohol sales (i.e. spirits) restricted to those aged 18 and over.

A short questionnaire was developed to examine: the time at which individuals had started drinking on the survey night; alcohol consumption up to the point of interview; whether they had preloaded (defined as drinking alcohol at their own or a friend's home before going out; participation in botellón was also recorded in Spain); expected alcohol consumption over the remainder of the night; whether they had, or intended to, use illicit drugs that night; and expected home time. The questionnaire was based on an existing tool used in UK drinking environments [18], adapted at a research meeting to be applicable in each location and ensure all questions were translated appropriately. A training package was delivered to research leads from each country to instruct them on consistent implementation of the study. Each research lead then recruited field researchers from their own city and repeated the training session with them.

In each city, researchers were instructed to identify peak periods for nightlife activity and to undertake data collection at these times in the streets around drinking venues popular with young people. The study took place on Thursday, Friday and Saturday nights (September to November 2010) between 10 pm and 5 am, with study timings dependent upon local nightlife activity. Recruitment of participants used a structured approach with teams of two researchers working in parallel and using a series of different locations in each city for periods of 1 h at a time. A target sample of 200 participants was set for each city (based on previous work in the UK [18]); the eligibility criteria was being a 16-35 year old drinker, using bars and nightclubs in the relevant city on the night of survey, and being a national of the survey country. To meet ethics requirements, researchers were instructed to visually assess potential participants and exclude those who were already too intoxicated to participate; the number of such individuals excluded ranged from three in Slovenia to 21 in Spain.

Researchers approached potential participants and asked them if they had time to complete a short anonymous survey about alcohol. Of 1,495 individuals approached, 483 refused to participate before the nature of the survey was explained to them and a further 131 declined after receiving a study explanation. Thus, overall compliance was 58.9% (Netherlands 66.8%, Slovenia 48.6%, Spain 55.4%, UK 69.3%). For the 881 individuals consenting, questionnaires were completed by researchers through an interview process. Following questionnaire completion, participants were breathalysed using the Lion 500 alcometer and results were recorded on their questionnaire. Completed questionnaires were returned to the UK and entered into a database for analysis using SPSS v17. At this stage, 43 questionnaires were excluded due to participants being outside of the target age or nationality range, questionnaires being incomplete or illegible, or data being clearly inconsistent, leaving a final sample of 838 (Netherlands n = 204; Slovenia n = 221; Spain n = 191; UK n = 222).

The questionnaire recorded alcohol use by detailing the number of standard and large drinks of lager/beer, cider, wine, alcopops and spirits participants had consumed by interview, and expected to consume over the remainder of the night. For analysis, drinks were converted to grams of alcohol using an online conversion tool [29]. To account for differences in alcohol strengths and serving sizes across sites, conversions were based on typical standard/large drink sizes and alcohol strengths in each country (with information obtained via research leads or published literature [30]). Thus, the gram value used for drink types varied between locations with, for example, a standard glass of wine coded as 16.8 g of alcohol in Slovenia and the UK, 11.2 g in Spain and 9.6 g in the Netherlands. For analysis, breath alcohol concentration was converted to the more commonly used blood alcohol concentration (%BAC; milligrams of alcohol per 100 ml of blood) according to established UK ratios [31]. Analysis used chi squared, Kruskal Wallace and logistic regression. Ethical approval for the study was obtained from Liverpool John Moores University research ethics committee in the UK.

## Results

A greater proportion of males were surveyed in the Netherlands, Slovenia and Spain, and younger samples obtained in Spain and the UK (Table 1). In each location, over three quarters of participants had started drinking at least 3 h before interview. Around half in the Netherlands and UK had been in the nightlife environment for < 3 h when interviewed, whilst most in Slovenia and Spain had been out for at least 3 h. Based on participants' expected home time, the Dutch sample reported the shortest expected total stay in the nightlife environment (59.5% < 5 h).

**Table 1:** Participant demographics and nightlife characteristics at the point of survey

		Netherlands	Slovenia	Spain	UK	P
	n	204	221	191	222	

Gender (%)	Male	60.3	59.7	64.7	46.8	
	Female	39.7	40.3	35.3	53.2	0.002
	n	204	221	187	222	

Age Group (%)	16-19	15.7	15.8	33.0	30.2	
	20-24	45.6	42.1	33.5	45.0	
	25-35	38.7	42.1	33.5	24.8	< 0.001
	n	204	221	191	222	

Hours since first drink at interview (%)	Less than 3 hours	20.8	24.9	22.9	24.8	
	3 to 5 hours	39.1	46.9	54.7	40.5	
	More than 5 hours	40.1	28.2	22.4	34.8	0.007
	n	192	213	170	210	

Hours in nightlife setting at interview (%)	Less than 3 hours	54.7	25.2	13.9	51.4	
	3 to 5 hours	25.8	46.3	53.9	28.1	
	More than 5 hours	19.5	28.5	32.2	20.5	< 0.001
	n	190	214	180	210	

Expected total hours in nightlife setting (%)	Less than 3 hours	17.4	4.2	0.7	4.2	
	3 to < 5 hours	42.1	34.1	30.3	42.1	
	5 to < 7 hours	22.1	28.5	40.1	23.1	
	More than 7 hours	18.5	33.2	28.9	30.6	< 0.001
	n	195	214	152	216	

Over half of Dutch (56.2%) and British (61.4%) participants had preloaded on the survey night. In Spain, 25.7% reported such preloading, yet a further 33.9% reported having participated in botellón (group drinking of off-licensed alcohol in public settings). As botellón can be a form of preloading in those attending bars and nightclubs, these Figures were combined to give the Spanish sample preloading levels similar to those in UK and Netherlands, with levels significantly lower in Slovenia (34.8%). Gender differences in preloading were only significant in the UK, where females reported higher levels than males (Table 2).

**Table 2:** Alcohol consumption and %BAC at interview and total expected alcohol consumption on the night out

		Females					Males				
		
		Netherlands	Slovenia	Spain	UK	P^a^	Netherlands	Slovenia	Spain	UK	P^a^
**% having preloaded** (including botellón in Spain^b^)		58.8	38.2	59.0	70.3	< 0.001	54.5	32.6	60.5	51.0	< 0.001

**Median grams of alcohol reported to have been consumed by interview**		54.4	50.4	50.4	56.8	0.147	92.8	64.0	70.4	104.0	< 0.001

**% having consumed drink type by interview**	Lager/Beer	55.6	32.6	21.2	16.1	< 0.001	85.4	56.1	40.5	75.0	< 0.001
	Cider	1.2	0	0	9.3	< 0.001	0.8	0	0	14.4	< 0.001
	Wine	60.5	46.1	22.7	28.8	< 0.001	9.8	25.8	9.1	7.7	< 0.001
	Alcopops	9.9	1.1	30.3	16.1	< 0.001	7.3	1.5	28.1	5.8	< 0.001
	Spirits	35.8	59.6	71.2	85.6	< 0.001	26.8	58.3	74.4	71.2	< 0.001

**% of grams of alcohol reported by overall sample accounted for by drink type^c^**	Lager/Beer	39.6	17.2	14.0	12.0		79.2	35.2	20.8	53.1	
	Cider	0.5	0.0	0.0	4.9		1.1	0.0	0.0	8.2	
	Wine	37.6	45.8	11.8	20.7		3.4	24.6	3.8	3.8	
	Alcopops	3.5	0.5	9.6	5.9		2.0	0.9	12.1	1.2	
	Spirits	18.8	36.5	64.7	56.5		14.3	39.3	63.3	33.6	

**Median %BAC^d ^at interview**		0.07	0.05	0.06	0.10	< 0.001	0.09	0.08	0.07	0.13	< 0.001

**% with BAC > 0.08%**		34.6	34.8	34.8	58.5	< 0.001	58.7	50.8	47.1	70.9	0.002

**Median expected grams of alcohol to be consumed over the remainder of the night^e^**		22.4	17.6	16.8	40.0	< 0.001	33.6	18.4	16.8	62.4	< 0.001

**Median grams of alcohol over whole night^f^**		76.8	66.4	72.0	104.8	< 0.001	139.2	79.2	87.2	176.8	< 0.001

**% expecting to binge drink^g^**		80.5	67.9	63.8	82.5	0.026	85.8	61.6	72.3	96.0	< 0.001

Statistics use chi squared and Kruskal-Wallis. Missing data: preloading n = 13; drinks types/grams of alcohol at interview n = 9; BAC n = 3.

^a^P between locations; ^b^Spain: males 24.4% preload, 36.1% botellón, females 27.9% preload, 31.1% botellón. ^c^Total grams of alcohol consumed by interview for individuals within each category were summed by drink type to show the proportion of grams reported by the sample that was accounted for by different drink types. ^d ^milligrams of alcohol per 100 millilitres of blood; legal driving limits are 0.08%BAC in the UK and 0.05% BAC in Netherlands, Slovenia and Spain. ^e ^Limited to the 691 individuals who were able to provide an estimate. ^f ^Including grams of alcohol consumed by interview and expected additional grams over the remainder of the night. ^g^Sum of grams consumed by interview and expected additional grams over the remainder of the night greater than 48.0 grams for females and 64.0 grams for males.

For females, there were no differences between nationalities in the median grams of alcohol participants reported having consumed by the point of interview (range 50.4 g to 56.8 g; Table 2). However, median BAC at interview varied significantly, being lowest in Slovenia (0.05%BAC) and highest in the UK (0.10%BAC). Among males, both reported grams consumed and BAC varied significantly, with both being highest in the UK (Table 2). In all countries, median grams of alcohol consumed by interview were significantly higher in males than females. Gender differences in BAC were significant in all countries except Spain.

**Figure 1:**
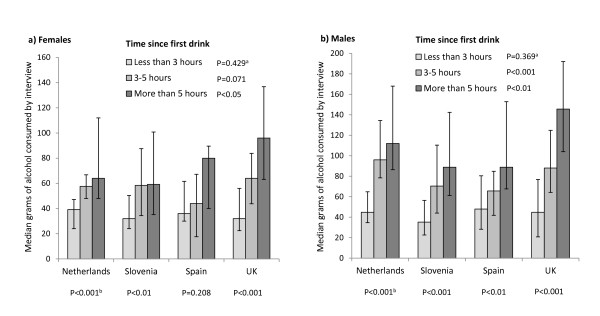
**Median grams of alcohol (and interquartile range) consumed by interview, by time since first drink**. Analysis uses Kruskal Wallis Test. ^a^P between nationalities across time periods; ^b^P between time periods within locations.

**Figure 2:**
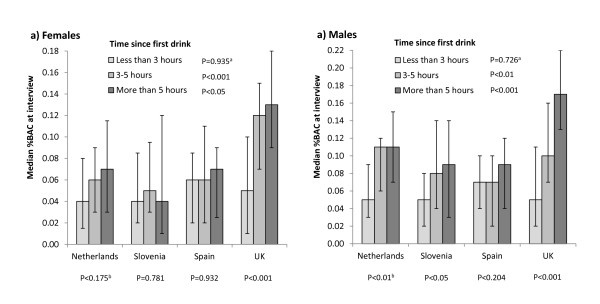
**Median %BAC (and interquartile range) measured at interview by time since first drink**. Analysis uses Kruskal Wallis Test. ^a^P between nationalities across time periods; ^b^P between time periods.

To account for varying interview times, alcohol consumption and BAC were analysed by the length of time between participants' first alcoholic drink that day and their survey participation (Figures 1 and 2). For both sexes, in the shortest time category (drinking < 3 h at interview) there were no significant differences between nationalities in median grams of alcohol consumed by interview or in BAC. There were also no gender differences within countries. In all samples, self-reported consumption by interview increased in those who had been drinking for longer, yet this increase was most pronounced in UK samples (Figure 1). Correspondingly, UK participants of both sexes measured significant increases in median BAC as length of time since the first drink increased (Figure 2). Males from the Netherlands and Slovenia also saw increases in BAC with time spent drinking, yet these increases were largely between the first and second time drinking categories. There were no differences in BAC by time drinking in females of other nationalities.

Analysis by interview hour found that median BAC in UK females exceeded 0.08% in those interviewed at 11.00-11.59 pm, and continued to increase up to the final UK survey hour of 2.00-2.59 am, where median BAC reached 0.16%. Similar increases were seen in UK males, with median BAC exceeding 0.10% at 11.00-11.59 pm and reaching 0.19% by 2.00-2.59 am. Among females of other nationalities, median BAC did not increase with interview time and reached 0.08% only in Dutch females surveyed at 2.00-2.59 am. In non-UK males, the highest median%BAC was seen in Slovenia at 2.00-2.59 pm (0.12%). The Spanish team interviewed latest into the night and here median BAC remained under 0.08% even in those surveyed from 4.00 to 5.00 am. Logistic regression was used to identify demographic and drink choice factors independently associated with high BAC (> 0.08%; the highest legal driving limit across the four study countries (UK) and a commonly used indicator of intoxication [32,33]). High BAC was associated with being male, aged > 19, British, having been drinking for longer at interview, and having consumed spirits prior to interview (Table 3).

**Table 3:** Adjusted odds ratios (AOR) for high blood alcohol concentration (> 0.08%BAC) at interview

		AOR	95%CIs	P
Gender	Female (Ref)			*
	Male	1.53	1.07-2.19	

Age Group	16-19 (Ref)			***
	20-24	2.50	1.66-3.78	
	25-35	1.96	1.28-3.01	

Country	Slovenia (Ref)			**
	Spain	0.89	0.54-1.46	
	Netherlands	1.17	0.75-1.83	
	UK	2.26	1.43-3.58	

Time spent drinking by point of interview	Less than 3 hours (Ref)			***
	3 to 5 hours	2.26	1.50-3.41	
	More than 5 hours	3.62	2.28-5.74	

Preloaded (or botellón)	No (Ref)			0.185
	Yes	1.25	0.90-1.73	

Consumed prior to interview:				

Lager/Beer	No (Ref)			0.227
	Yes	1.26	0.87-1.85	

Cider	No (Ref)			0.601
	Yes	1.30	0.49-3.46	

Wine	No (Ref)			0.700
	Yes	0.92	0.61-1.39	

Alcopops	No (Ref)			0.851
	Yes	1.06	0.60-1.86	

Spirits	No (Ref)			*
	Yes	1.59	1.07-2.34	

Had or intended to use illicit drugs on survey night	No (Ref)			0.876
	Yes	0.96	0.57-1.62	

*P < 0.05, **P < 0.01, ***P < 0.001; 95%CIs = 95% confidence intervals

Analysis uses logistic regression with all shown demographic and nightlife variables entered into the model. Missing data values limited the sample to 750.

Spirits were among the most common drinks reported by participants from the UK, Spain and Slovenia (Table 2). However, Dutch participants more commonly drank beer and, for females, wine. Among UK females and both sexes from Spain, spirits accounted for over half of all grams of alcohol consumed by interview. Beer accounted for the majority of grams consumed by Dutch males, and over half of those consumed by British males.

British participants expected to drink the most additional alcohol over the remainder of their night out, although differences between nationalities were only significant for males. These expected grams of alcohol were added to those participants reported having already consumed to give an estimate of total alcohol use on the survey night. For both sexes, this was highest in the UK, reaching a median of 104.8 g for females and 176.8 g for males. It was lowest in Slovenia, where median total grams remained < 70 g for females and < 80 g for males. In both Slovenia and Spain, total grams did not vary by gender. Median total grams in Dutch males was closer to that in British males (Table 2), yet in Dutch females median total grams was more akin to that in females from Spain and Slovenia. In all samples, consuming the total expected grams would have meant most participants drank enough to constitute binge drinking (here defined as drinking > 6 [female] or > 8 [male] UK units of alcohol in one session [11], equivalent to > 48 g of alcohol for females and > 64 g for males; range 61.6% of males in Slovenia to 96.0% of males in the UK).

In addition to alcohol consumption, 10.7% of the sample reported having used, or intending to use, illicit drugs on the night of survey, predominantly cannabis (73.3%) followed by cocaine (30.2%). Drug use was most commonly reported by Spanish participants (21.3%, compared with 6.0%, 8.1% and 9.0% in the Dutch, British and Slovenian samples respectively).

## Discussion

Our study faced a number of limitations beyond those common to nightlife research [18,34]. Differences in nightlife habits meant that interviews were not conducted at consistent times in each study site but rather were targeted at the busiest periods in each city. Thus, we did not attempt to recruit representative samples of nightlife users but rather prospective samples indicative of the range of individuals participating in recreational drinking at peak times. With study implementation limited to one city in each country, sample sizes relatively small and overall compliance at 58.9%, findings should only be extrapolated with caution.

Recording alcohol consumption across four cultures was complicated by variations in alcoholic drink types and measures. Thus, we recorded the numbers of different drinks individuals reported in two size categories and converted these to grams of alcohol based on local standard drink sizes and strengths. The reliability of alcohol estimates will be further constrained by variations in self-poured alcohol consumed during preloading [35] and recall issues [34]. Studies suggest that heavier drinkers can be more likely to underestimate their alcohol consumption [36,37], an effect potentially seen among UK participants in our study. Thus, UK females reported a similar median of grams of alcohol at interview to females of other nationalities but had significantly higher median BAC, suggesting that alcohol use may have been under-reported (Table 2). The use of breathalysers provided a mechanism for recording a more comparable measure of intoxication across cities, although the same BAC can have different effects on different people and consequently BAC is not a reliable measure of drunkenness [34]. Further, individuals who were severely intoxicated were excluded to meet ethical requirements, meaning median reported alcohol consumption and BAC are likely to be underestimated. Despite training researchers in identifying such individuals for exclusion, differences in researchers' cultural and personal perceptions of intoxication may have introduced further bias to the samples obtained. However, even in Spain where the number excluded was highest, such individuals represented only 5.8% of potential participants. Finally, the validity of responses to questions on expected behaviours (alcohol use, home time) post survey could not be verified in our study, and in particular several participants (n = 147, 17.5%) felt unable to provide an estimate of how much additional alcohol they would consume that night.

This investigation into drinking behaviours in European nightlife found high levels of alcohol consumption in all cities. The majority of individuals reported consumption equivalent to levels commonly used to measure binge drinking [11], and by the point of interview median reported alcohol consumption had already reached binge drinking levels for both sexes, with many individuals intending to continue drinking. Despite screening out those assessed as too drunk to participate in the survey, 56.2% of all males and 42.7% of all females had BACs greater than 0.08%. However, significant differences were seen between nationalities in most measures of alcohol use. Specifically, participants from the UK had significantly higher BACs at interview and expected to consume the greatest total grams of alcohol across the course of their night out (Table 2). In line with the characterised north-south divide in drinking cultures in Europe [10], the lowest BACs and expected consumption levels were seen in the southern countries (Slovenia and Spain). In all countries, reported alcohol use and BAC were higher in males than in females, although differences were not always significant.

A range of studies have documented the high levels of alcohol use and intoxication in UK nightlife users [18,23]. Cross-national studies have suggested they are also at increased risk of social harms associated with their drinking, particularly violence [11]. This culture of intoxication is clearly apparent in our study, with findings indicating that many UK nightlife users fail to moderate their drinking across a night out. Thus, whilst BAC and grams of alcohol consumed by interview in individuals from the Netherlands, Slovenia and Spain increased more moderately or not at all with the time spent drinking, in UK participants both continued to increase on a substantially steeper trajectory. Further, median %BAC increased with interview time in UK respondents, with both genders exceeding a median of 0.08%BAC by the 11.00-11.59 pm time period and reaching at least double this level by 2.00-2.59 am. Notably, median BAC at interview was higher in UK females than that of males of other nationalities. These findings are consistent with UK nightlife being a considerably more intoxicated environment than that in other European countries.

There is a growing evidence base on the effectiveness of strategies to reduce alcohol-related harm in drinking environments. Studies suggest that measures including targeted enforcement in venues associated with alcohol-related problems and bar staff training can be effective, particularly when integrated into broader, multi-agency programmes that mobilise communities into co-ordinated action [38,39]. Regulatory measures that reduce the availability of alcohol through, for example, restrictions on alcohol outlet density and increased alcohol price, are also likely to have significant effect but are rarely used in practice [40]. However, most evidence on the effectiveness of interventions in drinking environments stems from studies outside of Europe (North America and Australia), with European evidence limited largely to studies in northern European countries (UK and Scandinavian countries [9,38]). These interventions are often designed to reduce alcohol-related crime and violence in cultures of intoxication. Such interventions may not be appropriate to tackle the smaller proportion of individuals drinking heavily in other countries. A study of nightlife risk behaviours in young Europeans suggested that drink driving may be a particular issue for those from southern countries [12,41].

Strategies to reduce alcohol-related harm in drinking environments should not limit their attention to on-licensed alcohol consumption. Preloading has been identified as a major feature of nightlife participation in several countries [36,42-44], and has been associated with greater alcohol consumption in nightlife users as well as involvement in violence [23,44]. No independent associations were found between preloading and high BAC in our study, although preloading was found to be widespread. Over half of participants of both genders from the UK, Netherlands and Spain, and a third from Slovenia, had consumed alcohol before going to bars and nightclubs on the survey night. Gender differences in preloading were only significant in the UK, where females reported higher levels than males. Preloading can be undertaken for a variety of reasons including to save money (through consumption of cheaper off-licensed alcohol), to achieve drunkenness and to socialise with friends. This latter reason may be particularly important for British females, for whom the act of getting ready to go out is often a protracted social process that can itself form a key part of the night out [45]. Although the drivers behind female preloading in the UK have yet to be fully explored [46], factors around safety, confidence and group bonding may also be important for young women preparing to visit an environment perceived as sexually and physically aggressive. These issues require further research, as do the lower levels of preloading identified among the sample from Slovenia.

In Spanish participants, around half of those classed as preloaders had participated in botellón, a phenomenon of drinking in public places that has become increasingly widespread among Spanish youth and is appearing in other southern European countries (e.g. Portugal [47]). Like preloading in other settings (e.g. homes, college residences [43]), a key reason for participation in botellón is the lower price of off-licensed alcohol compared with that in bars and nightclubs [26]. Preloading with off-licensed alcohol can mean that bars and nightclubs face an increasingly intoxicated customer base, which is likely to hamper strategies that aim to reduce harm in drinking environments through, for example, promoting responsible beverage service and bar management.

Studies of the Spanish botellón and youth drinking elsewhere in Europe are noting a shift in the type of alcohol consumed by young people in southern Europe, characterised by greater beer and spirits consumption [14,48]. Across Europe in general, traditional preferences (particularly among males) for wine in southern countries and beer or spirits in northern countries [4,10] have diminished in recent years, although wine and beer continue to account for the greatest share of alcohol use in most southern and northern countries respectively. In 2002, spirits were found to account for less than a third of alcohol consumption across each of the 15 European Union countries and Norway [4]. In contrast, our study of young adults in nightlife environments found a preference towards spirits in both sexes from all participating countries except the sample from the Netherlands. In particular, over 70% of Spanish participants had consumed spirits prior to interview, with spirits accounting for almost two thirds of all grams of alcohol consumed by the Spanish sample. Although spirits are often consumed during botellón [48], spirits consumption was most common in those who had only consumed alcohol in bars and nightclubs (83.1%, compared with 64.4% of botellón participants and 74.5% of pre-drinkers, *P *< 0.05). Spirits increase BAC more rapidly than consumption of other drink types [49] and have been associated with greater alcohol consumption, more frequent risky drinking occasions, and drinking motives focused on fun and drunkenness [50]. In our study, consumption of spirits was independently associated with having a BAC > 0.08% at interview. The strong preference for spirits in our samples thus provides support for a growing normalisation of intoxication among young Europeans.

Drinking behaviours in European nightlife settings will be influenced by a range of social, cultural, economic and environmental factors, including the availability and affordability of alcohol. For example, although findings are mixed, studies suggest that greater density of alcohol outlets and longer opening hours are associated with increased alcohol use and related harms [51,52]. The price of alcohol also has a strong influence, particularly in young people, and studies show that alcohol has become more affordable in most European countries over recent years [53]. However, national economic analyses say little about local conditions, and factors including cheap drinks promotions in nightlife venues and large discrepancies between on and off licensed alcohol prices will impact on how and where young people drink over the course of a night out. Equally, the drinking environment in licensed premises (e.g. crowding, poor cleanliness), bar manager and staff practice (e.g. service of alcohol to drunk customers), and local alcohol policy and enforcement activity (e.g. policing of problem premises, punishment of sales of alcohol to minors) may all affect drinking behaviours and alcohol-related harm [22,38]. There is currently a lack of data on such factors within different European drinking environments and whether they are driving the differences in drinking patterns identified in this study.

## Conclusions

This study has provided an examination of drinking behaviours and BAC among young people in four European drinking environments. High levels of preloading and alcohol use were seen in nightlife in all cities. Excessive alcohol use can lead to a wide range of health and social harms, including violence and unintentional injury, risky sexual behaviour, anti-social behaviour, and poor educational and work performance. Critically, occasional heavy drinking sessions also increase an individual's risk of later mortality through an alcohol-related disease [54]. With alcohol already a leading cause of death in young Europeans [55], the high levels of alcohol use seen in our study emphasise the need for co-ordinated strategies to reduce harm in drinking environments. However, while a wide range of drinking patterns was present in all cities studied, those in Spain, Slovenia and the Netherlands were largely characterised by steady, more moderate intoxication compared with escalating inebriation in the UK. As drinking patterns, alcohol policy and commercial interests converge across Europe, the challenge for public health is to ensure that models of escalating inebriation in nightlife environments are replaced rather than replicated.

## Endnotes

^1^Data on drinking environments were obtained by research partners from the relevant authorities in each city.

## Competing interests

In the past 5 years, Centre for Public Health received a grant from Drinkaware to undertake an independent study of drinking behaviours among students and MAB has provided them with independent medical advice. Drinkaware is an independent UK-wide charity supported by voluntary contributions from the alcohol and supermaket industries.

## Authors' contributions

KH designed the study, participated in data collection, analysed the data and wrote the manuscript. ZQ co-designed the study, managed study implementation, participated in data collection, and edited the manuscript. MB contributed to study design, data analyses and writing of the manuscript. NvH, AC, MK, MJ, MD and LV contributed to study design, study implementation and data collection and edited the manuscript. All authors read and approved the final manuscript.

## Pre-publication history

The pre-publication history for this paper can be accessed here:

http://www.biomedcentral.com/1471-2458/11/918/prepub
